# Oxytocin attenuates neural response to emotional faces in social drinkers: an fMRI study

**DOI:** 10.1007/s00406-020-01115-0

**Published:** 2020-02-19

**Authors:** Patrick Bach, Anne Koopmann, Jan Malte Bumb, Sina Zimmermann, Sina Bühler, Iris Reinhard, Stephanie H. Witt, Marcella Rietschel, Sabine Vollstädt-Klein, Falk Kiefer

**Affiliations:** 1 Department of Addictive Behavior and Addiction Medicine, Central Institute of Mental Health, Medical Faculty Mannheim/Heidelberg University, J5, 68159 Mannheim, Germany; 2 Feuerlein Center on Translational Addiction Medicine (FCTS), University of Heidelberg, Heidelberg, Germany; 3 Department of Biostatistics, Central Institute of Mental Health, Medical Faculty Mannheim/Heidelberg University, Heidelberg, Germany; 4 Department of Genetic Epidemiology in Psychiatry, Central Institute of Mental Health, Medical Faculty Mannheim/Heidelberg University, Heidelberg, Germany

**Keywords:** fMRI, Oxytocin, Alcohol use disorder, Faces, Emotion, Craving

## Abstract

**Introduction:**

Oxytocin is a key mediator of emotional and social behavior that seems to be of relevance for the development and maintenance of addictive behaviors. We thus investigated the effect of oxytocin on neural response and behavior during a face-matching task in a sample of social drinkers.

**Methods:**

Thirteen social drinkers underwent a randomized double-blind placebo-controlled cross-over functional magnetic resonance imaging face-matching task with and without prior intranasal application of 24 international units oxytocin. Effects of oxytocin and task condition (faces, shapes) on brain activation and individual task performance were assessed.

**Results:**

Face-matching compared to shape-matching trials resulted in higher brain activation in the bilateral amygdala, hippocampus and parts of the occipital gyri. Oxytocin application vs. placebo reduced activation in bilateral amygdala, parts of the frontal gyri, and the parietal lobe. Region of interest analyses indicated that the oxytocin-induced attenuation of amygdala response was specific to face-stimuli and associated with lower subjective alcohol craving, and a lower percentage of heavy-drinking days (defined as ≥ 5 standard drinks/day).

**Conclusion:**

For the first time, we could show that a larger oxytocin-induced attenuation of amygdala response to fearful faces is associated with lower subjective craving for alcohol and percentage of heavy drinking days in social drinkers. Modulation of amygdala activation, induced by emotional stimuli, might represent a neurobiological substrate of oxytocin’s protective effects on drug seeking behavior.

**Electronic supplementary material:**

The online version of this article (10.1007/s00406-020-01115-0) contains supplementary material, which is available to authorized users.

## Introduction

There is a growing body of evidence supporting the role of the brain oxytocin system in addiction. Preclinical studies demonstrated that oxytocin administration reduced alcohol self-administration in rats and mice models of alcohol addiction [14, 18, 24]. Preliminary clinical studies also showed that oxytocin reduces withdrawal symptoms and alcohol craving in patients with alcohol use disorder (AUD) [20, 23]. However, studies yielded inconsistent results and research on oxytocin’s effect on social cognition indicated that context and individual predispositions might be relevant factors that mediate differential effectivity. Regarding studies on addictive phenotypes, it was shown that attenuation of anxiety might be a relevant factor that mediates the effects of oxytocin on addictive behavior in animals [21]. Studies showed that oxytocin exposure in adolescent rats resulted in persistent reductions in anxiety and alcohol consumption [2]. A study in humans with AUD demonstrated that oxytocin yielded significant alcohol craving reductions only in those with anxious attachment style [20]. The idea that oxytocin might act on addictive phenotypes—in part—via attenuation of anxiety is also compatible with the body of evidence demonstrating that central oxytocin modulates social behavior and regulates anxiety, fear, and stress perception [31]. Hence, the effects of oxytocin on emotion-processing and their relation to alcohol craving are of interest with regards to uncovering the neurobiological basis of oxytocin’s effects on addictive phenotypes. A review of the effects of oxytocin on brain activation during functional magnetic resonance imaging (fMRI) based investigation of the neural response to facial emotions showed that oxytocin attenuates response to negative facial emotions most reliably in the bilateral amygdala [31], whereas effects in other brain regions were reported less consistently.

The recent parent study of presented work incorporated a sample of social drinkers in addition to animal data and human post-mortem data. Results demonstrated that oxytocin-application compared to placebo reduced neural alcohol cue-reactivity in the group of social drinkers in the insula, hippocampus, parahippocampus, cingulate gyrus, the inferior and the medial frontal gyrus, and in visual and motor regions. Further, pharmacological oxytocin administration reduced cue-induced re-instatement of alcohol-seeking in the animal sample of the study.

To date, there is very few data on the neurobiological basis of the potential link between oxytocin’s effects on the processing of social stimuli, anxiety on the one hand and addictive behavior on the other hand. Hence, we set out to explore oxytocin’s effects on the neural and behavioral response to negative social emotional visual stimuli during an fMRI “Faces” task [9] and their associations to craving and alcohol consumption in a sample of social drinkers.

## Methods

### Study design and assessment

The study was a randomized, double-blind, placebo-controlled, cross-over study (clinical trial number DRKS00009253), investigating the effects of intranasal oxytocin application (24 international units, IU) on brain activation and clinical measures (i.e. craving) in 18 non-treatment seeking heavy social male drinkers [8]. All subjects underwent two separate experimental sessions at an interval of two weeks, comprising the assessment of clinical measures and two fMRI scans. Participants randomly received oxytocin either before the first or before the second session. The ethics committee of the University of Heidelberg approved all experimental procedures. At the first assessment day, all participants provided demographic information and completed the standardized psychiatric interview (SCID), to rule out any axis one disorder, other than nicotine addiction. At both investigation days, all participants underwent drug urine screening and breath alcohol level measurement. Prior to the scanning session, participants completed a series of clinical scales and questionnaires, such as the Beck Depression Inventory (BDI [10]), the perceived stress scale (PSS [4]), the Obsessive Compulsive Drinking Scale for Alcohol Dependence (OCDS [1]) and the Fagerstroem Test for Nicotine Dependence (FTND [11]), as well as the State Trait Anxiety Inventory (STAI [29]). Substance use patterns during the 90 days before the experiment were assessed using a short semi-structured interview at the beginning of the second experimental day (Form 90 [27]).

A single dose of 24 IU oxytocin vs. placebo was administered as 12 spray puffs (6 puffs in each nostril) 45 min prior to the MRI measurement. The functional MRI tasks were performed about 60 min after oxytocin application. This timeframe was chosen according to the results of previous work, indicating regional cerebral blood flow changes between 25 and 78 min after oxytocin application [22]. Participants underwent an fMRI measurement comprising a face perception task [9] and an alcohol cue-reactivity task [32], as well as a structural measurement. Results of the alcohol cue-reactivity task are reported in the parent study [8].

### Study sample

A sample of 18 male social drinkers was recruited via advertisements in local newspapers, bulletins at public institutions as well as via social networks. A total of 15 participants attended the scheduled appointment and completed the first scanning session. One participant did not attend the second scanning session, leaving 14 participants that successfully completed the experimental procedure. One participant had to be excluded from further analyses, due to heavy movement in the scanner, leaving datasets of 13 participants for statistical analyses (see supplementary figure S1 for CONSORT flow chart). All participants were required to be aged 18–65 years and meet the definition of “social drinking” (i.e. alcohol consumption ≥ 1 standard drink, defined as 12 g alcohol, on at least 2 days per week), as well as being right-handed, and having normal vision. Subjects that (1) experienced severe withdrawal symptoms in the past, or (2) underwent inpatient treatment, due to alcohol intoxication in the past, or (3) met the criteria for any other axis-I disorder, except from nicotine addiction in the last 12 months (according to DSM-IV), or (4) took any psychoactive substances, anti-craving or anticonvulsive medication within the last months, or (5) had any comorbid severe internal or neurological condition, or (6) had a positive drug-screening, or (7) had any contraindications for receiving a MRI-scan (e.g. tattoos, metal implants, pacemakers), or (8) had contraindications to the administration of oxytocin, or (9) had positive breath alcohol levels (> 0), were excluded from the study. Breath alcohol was controlled on both days of scanning and no subject had a breath alcohol content > 0. In addition, unheralded drug urine screenings were conducted on both experimental days and no participant had a positive screening for any substance.

### fMRI face task

During the fMRI task that was adapted from Hariri et al. [9], participants were presented either faces or shapes and had to decide which of two faces (respective shapes) presented on the bottom of the screen match the face (respective shape) at the top of the screen. The faces depicted either angry or fearful expressions. Participants were instructed to indicate the matching shape or face via press of a button (left vs. right). Reaction times and correct vs. incorrect button presses were recorded during the experiment. The entire task consisted of eight blocks (i.e. 24 shape- and 24 face-trials, amounting to 48 trials in total) and took approximately six minutes in total.

### fMRI acquisition, pre-processing and statistical analyses

The fMRI measurement was conducted using a three Tesla whole-body-tomograph (MAGNETOM Trio, TIM technology, Siemens, Erlangen, Germany). While participants performed the faces task a total of 134 T2*-weighted echo-planar images (EPI) were acquired using a TR (repetition time) of 2 s, a TE (echo time) of 30 ms, a flip angle of 80°, 28 slices, slice thickness = 4 mm, 1-mm gap, voxel dimensions 3 × 3 × 5 mm^3^, FOV = 192 × 192 mm^2^, 64 × 64 in-plane resolution). To reduce artifacts due to magnetic saturation effects, the first five scans were excluded from analyses. All MRI data were pre-processed using the statistical parametric mapping software for Matlab (SPM, Wellcome Department of Cognitive Neurology, London, UK) version 8. Image data were temporally realigned to minimize temporal differences in slice acquisition, corrected for residual geometric distortion on the basis of the acquired magnetic field map, spatially realigned, corrected for micro-movements and normalized to a standard MNI (Montreal Neurological Institute, Quebec, Canada) EPI template. Subsequently, images were smoothed using an isotropic Gaussian kernel for group analysis (8 mm Full Width at Half Maximum). For every participant, first level statistics were computed, modelling the different experimental conditions (faces vs. shapes) in a general linear model (GLM) and adding movement parameters as nuisance variables in GLM standard procedure, according to SPM manual and previous studies on this paradigm [15]. Resulting contrast images for the contrasts “faces” and “shapes” were imputed in a second-level full factorial model with the factors medication (oxytocin vs. placebo) and condition (faces vs. shapes). The succession of scans (i.e. whether participants received oxytocin during the first or second scan) was included as intervening factor to control for sequence-effects. To control for multiple comparisons, a combined voxel-wise and cluster-extent threshold, corresponding to a family wise error (FWE) rate of *p*_FWE_ < 0.05, was determined using the AlphaSim module of the NeuroElf toolbox (www.neuroelf.net) for Matlab. For a pre-set voxel-wise threshold of *p* < 0.001, the AlphaSim procedure determined a cluster extent threshold of 52 voxels (10,000 Monte Carlo simulations, estimated smoothness based on the residual images was *x* = 12.33 mm, *y* = 12.19 mm, *z* = 11.12 mm). All thresholds for the fMRI analyses were set accordingly. In accordance to our strong a priori hypothesis for the amygdala, we conducted region-of-interest (ROI)-based analyses for this region in addition to our whole-brain analyses using the small volume correction (SVC) function of SPM that restricted the search area to the standardized anatomical amygdala masks from the Wake Forest University PickAtlas (WFU PickAtlas; https://www.fmri.wfubmc.edu/downloads). We applied a voxel-wise threshold within the volume of interest of *p*_FWE_ < 0.05 (two ROI analyses in total). It should be noted that this approach is less stringent than the whole-brain correction of fMRI activation, but still seems reasonable with regards to the strong a priori hypothesis on the amygdala as regions of interest. In addition, we followed established recommendations on performing small volume correction, e.g. the ROIs were determined independently of the specific test on which the correction is performed, using standardized anatomical masks [25]. To perform additional association and factorial analyses, functional brain activation was extracted using the standard functions included in the MarsBar software package (https://marsbar.sourceforge.net/) and standard anatomical masks from the WFU PickAtlas) for (1) the left and (2) right amygdala. Data were exported to IBM SPSS Statistics version 24.0 for following analyses. Firstly, repeated measures analyses of variance with the factors task-condition and medication were applied to test effects of oxytocin vs. placebo on functional brain activation in the left and right amygdala ROI during the “faces” and “shapes” conditions. For the repeated measures analyses of variance, we assessed the assumption of normality by visual inspection of value distribution of the respective variables and additionally using the Shapiro–Wilk's test for normality. Further, Mauchly’s test for sphericity (as implemented in IBM SPSS Statistics) was used to test the assumption of sphericity. Secondly, we tested associations between functional activation in the left and right amygdala ROIs, specifically the oxytocin-related modulation of functional activation (contrast: “placebo—oxytocin”), and drinking data (1—mean alcohol consumption during the last 90 days, 2—percent of heavy drinking days) and psychometric data (3—OCDS sumscore) by applying bivariate correlation analyses.

### Analyses of demographic, clinical and performance data

Data analyses were performed using SPSS version 24.0. Paired *t* tests were applied to analyze differences in clinical characteristics (i.e. BDI scores) between scanning sessions. Repeated measures analyses of variance, controlling for sequence-effects, were used to investigate effects of oxytocin on task performance during the MRI face task. Statistical significance level was set to an alpha of 0.05.

## Results

### Sample characteristics and task performance

A total of 13 participants (mean age 34.5 years, SD 16.7; mean weight 83.35 kg, SD 10.9) provided valid fMRI data for the placebo and the oxytocin condition. On average, participants drank 34 g alcohol (SD 21.6 g, range 10–73 g) per day that amounted to 2.86 (SD 1.8, range 0.8–6.1 drinks) standard drinks of alcohol (à 12 g) per day. On a drinking day, participants drank on average 5.3 (SD 2.3, range 2.0–9.6 drinks) standard drinks with an average of 21.6% heavy drinking days (%HDD, SD 27.9, range 0–100%). Participants reported a high percentage of drinking days (52.5%, SD 24.4, range 27–100%). This indicates that the current sample reflects a group of moderate social drinkers (i.e. defined as one or more drinks per occasion during the last 3 months and more than once a year) [30]. Two participants were smokers (15% of sample) with an average FTND score of 2.0 (SD 2.8). Psychometric scores for the first and second investigation day are depicted in Table 1. The comparison of task performance data between trials with and without oxytocin application revealed no significant differences in response times and accuracy rates to faces or shapes (*p*_min_ = 0.116).

**Table 1:** Demographic, clinical data, and task performance data, expressed as means and standard deviations (SD)

Social drinker (*n* = 13)	Mean (SD)	Mean (SD)	Statistics	Significance
Clinical scales	First scan	Second scan		
OCDS (sumscore)	7.2 (3.8)	7.2 (4.2)	*t*_(12)_ = 0.210	*p* = 0.837
STAI (trait sumscore)	32.9 (6.9)	31.0 (6.9)	*t*_(12)_ = 1.345	*p* = 0.204
PSS (sumscore)	6.1 (4.5)	6.9 (3.6)	*t*_(12)_ = 1.046	*p* = 0.316
BDI (sumscore)	5.4 (2.7)	4.2 (4.1)	*t*_(12)_ = 2.004	*p* = 0.068

Social drinker (*n* = 13)	Mean (SD)	Mean (SD)	Statistics	Significance
Performance data	Oxytocin	Placebo		
Accuracy during faces condition (%)	99.0 (3.5)	98.4 (2.7)	*F*_(1,11)_ = 1.634	*p* = 0.277
Reaction time during faces task (ms)	1277.6 (343.2)	1228.4 (373.8)	*F*_(1,11)_ = 2.174	*p* = 0.116
Accuracy during shapes condition (%)	94.6 (8.2)	98.1 (2.2)	*F*_(1,11)_ = 0.083	*p* = 0.779
Reaction time during shapes task (ms)	1081.0 (257.5)	1099.1 (334.8)	*F*_(1,11)_ = 0.498	*p* = 0.495

*BDI* Beck Depression Inventory, *OCDS* Obsessive–Compulsive Drinking Scale, *PSS* perceived stress scale, *STAI* State-Trait-Anxiety Inventory, *SD* standard deviation, *t* *t* statistic, *F* *F* statistic

*Significant differences *p* < 0.05

### fMRI brain activation

**Fig. 1:**
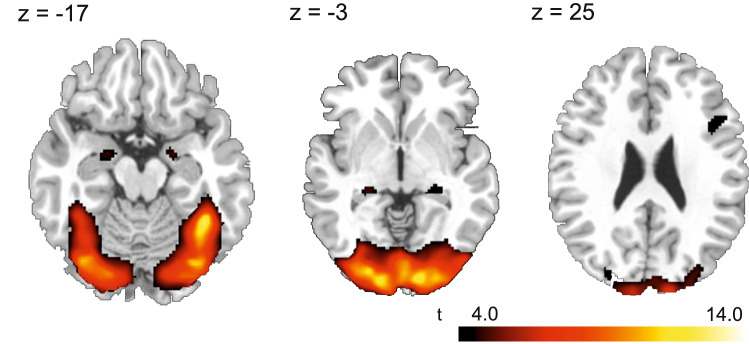
Depiction of brain areas that show a higher activation during processing of faces compared to shapes (contrast: “faces—shapes”, *n* = 13, *t* values ranging from 4 to 14 in all three slices, height-threshold: *p* < .001, extent-threshold: cluster size ≥ 52 voxel, corresponding to *p*_FWE_ < 0.05)

Analyses of imaging data indicated significant main effects of task condition (faces vs. forms) and medication (oxytocin vs. placebo) on neural brain activation in limbic and cortical brain areas. In line with previous work, face stimuli relative to shapes induced higher brain activation in a cluster of brain areas that included the bilateral amygdalae, the hippocampus, thalamus, the fusiform gyrus and parts of the parietal, occipital and temporal gyri (see Fig. 1; Table 2). Compared with the placebo condition, oxytocin reduced neural activation in parts of the superior, middle and inferior frontal gyri, the pre- and postcentral gyri, precuneus, as well as in the angular gyrus (see Table 2).

**Table 2:** Brain areas that show significant condition- (faces vs. shapes) or trial-dependent (oxytocin vs. placebo) differences in neural activation (*n* = 13, combined voxel-wise- [*p* < 0.001] and cluster-extent-threshold [*k* > 52 voxel], corresponding to *p*_FWE_ < 0.05)

Side	Lobe	Brain regions	Cluster size	MNI coordinates (*x*, *y*, *z*)			*t*_max_
(a) Faces > shapes							
L & R	Temporal, Occipital, Parietal	Lingual Gyrus, Cuneus, Middle Occipital Gyrus, Fusiform Gyrus, Inferior Occipital Gyrus, Parahippocampal Gyrus, Inferior Temporal Gyrus, Superior Occipital Gyrus, Middle Temporal Gyrus, Cerebellum	14,623	14	− 102	8	14.93
L		Hippocampus	60	− 24	− 30	− 4	4.45
R		Amygdala, Parahippocampal Gyrus	67	20	− 6	− 16	4.44
L		Amygdala, Hippocampus	95	− 24	− 6	− 20	4.35
R		Thalamus	68	24	− 30	− 2	4.12
R	Frontal	Inferior Frontal Gyrus, Precentral Gyrus	204	42	20	20	4.04
(b) Faces < shapes							
–	–	–	–	–	–	–	–
(c) Oxytocin < placebo							
L	Frontal	Precentral Gyrus, Middle Frontal Gyrus, Postcentral Gyrus, Frontal Eye Field	162	− 50	− 2	48	5.11
L & R	Frontal	Superior and Middle Frontal Gyrus, Supplementary Motor Area, Frontal Eye Field	745	20	14	66	4.92
L	Frontal	Precentral Gyrus, Superior and Middle Frontal Gyrus	118	− 26	− 12	44	4.77
L	Parietal	Precuneus, Superior Parietal Gyrus	72	− 20	− 66	62	4.32
L	Parietal	Angular Gyrus, Superior and Middle Parietal Gyrus	136	− 36	− 62	48	4.17
L	Frontal	Middle and Inferior Frontal Gyrus	64	46	28	28	4.00
L		Amygdala*	127	− 26	− 4	− 22	3.57
R		Amygdala*	180	20	− 4	− 16	3.73
(d) Oxytocin > placebo							
–	–	–	–	–	–	–	–

*t*_max_ = maximum *t* value

^*^Region of interest (ROI)-based small volume corrected (SVC) analyses: ROIs for the left and right amygdala were defined by standard anatomical masks from the Wake Forest University (WFU) PickAtlas; *p*_FWE_ = 0.013 for the left amygdala and *p*_FWE_ = 0.010 for the right amygdala (*p*_FWE_ = family wise error rate)

**Fig. 2:**
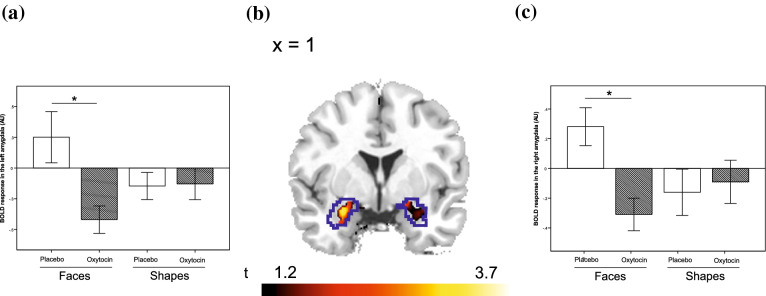
Depiction of oxytocin-induced attenuation of blood oxygenation level dependent (BOLD) response during presentation of faces in the **a** left and **c** right amygdala ROIs, presented as **b** rendering on a coronal slice (contrast: “faces”, *n* = 13, boundaries of amygdala region of interest (ROI) mask from the Wake Forest University (WFU) atlas are shown in blue, *p*_FWE_ < 0.05 voxel-wise threshold for the limited ROI volume)

Additional small volume corrected ROI-based analyses of the full factorial model confirmed that oxytocin also significantly reduced activation in the left and right amygdalae (contrast: oxytocin < placebo, see Table 2; Fig. 2). In addition, repeated measures analyses of variance of extracted amygdala functional brain activation demonstrated that the oxytocin effect on amygdala activation was specific to the face-matching condition, i.e. data showed a significant interaction effect (medication × task-condition) on functional brain activation in the left (*F*_leftAmygdala(1,12)_ = 19.52, Eta^2^ = 0.62 *p* = 0.001) and right amygdala (*F*_rightAmygdala(1,12)_ = 14.90, Eta^2^ = 0.55 *p* = 0.002) (see Fig. 2).

### Associations between neural activation, behavioral, and performance data

**Fig. 3:**
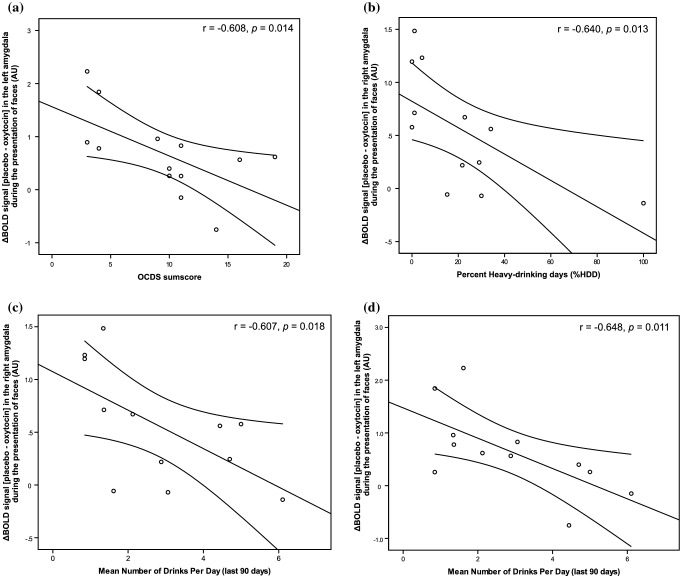
Scatterplots depicting the negative associations between **a** the attenuation of blood oxygenation level dependent (BOLD) response in the left amygdala during the face-matching trials specified in arbitrary units (AU) (i.e. larger difference in neural activation between oxytocin and placebo trials during face-matching trials [contrast: faces, placebo—oxytocin]) and Obsessive–Compulsive Drinking Scale (OCDS) scores (*r* = − 0.608, *R*^2^ = 0.369, *p* = 0.014), **b** between the attenuation of blood oxygenation level dependent (BOLD) response in the right amygdala during the face-matching trials specified in arbitrary units (AU) and the percentage of heavy-drinking days (*r* = − 0.640, *R*^2^ = 0.409, *p* = 0.013) and between **c** right amygdala activation during the face-matching and the mean alcohol consumption during the last 90 days (*r* = − 0.607, *p* = 0.018, *p*_FDR_ = 0.036) and **d** between the left amygdala activation and the mean alcohol consumption during the last 90 days (*r* = − 0.648, *p* = 0.011, *p*_FDR_ = 0.036)

Regarding the associations between functional brain activation and the three predefined parameters (1—mean alcohol consumption during the last 90 days, 2—percent of heavy drinking days and 3—OCDS sumscore), there was a significant negative correlation between the attenuation of left and right amygdala activation during the face-matching trials (i.e. larger difference in neural activation between oxytocin and placebo trials during face-matching trials [contrast: faces, placebo—oxytocin]) and the mean alcohol consumption during the last 90 days for the left (*r* = − 0.648, p = 0.011 *p*_FDR_ = 0.036) and right (*r* = − 0.607, *p* = 0.018, *p*_FDR_ = 0.036) amygdala (see Fig. 3). Additionally, the extent of brain response attenuation by oxytocin during face-matching trials in the left amygdala significantly correlated with the OCDS total score (*r* = − 0.608, *p* = 0.014, *p*_FDR_ = 0.036), i.e. a larger oxytocin-induced reduction of amygdala activation during face-matching trials was associated with lower craving scores (see Fig. 3). The association between oxytocin-induced attenuation of neural activation in the right amygdala and OCDS scores was not significant (*r* = − 0.342, *p* = 0.151, *p*_FDR_ = 0.152). Further, we found a significant negative association between the oxytocin-associated attenuation of right (*r* = − 0.640, *p* = 0.013, *p*_FDR_ = 0.036), but not left (*r* = − 0.449, *p* = 0.071, *p*_FDR_ = 0.095) amygdala activation during face-matching trials and the percentage of heavy-drinking days, which were defined as ≥ 5 standard drinks/day (see Fig. 3). Importantly, the effect was specific to the face matching condition. Data showed that there was no significant association between neural activation during the shape-condition and above states measures (all *p* values > 0.05).

**Fig. 4:**
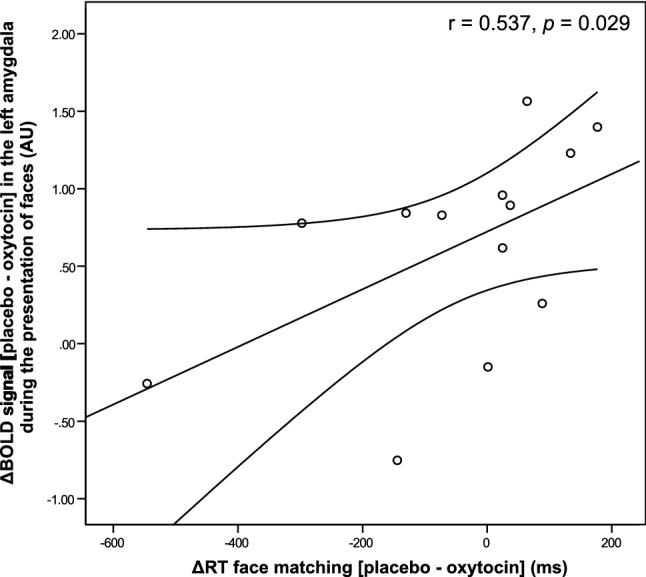
Scatterplot depicting the positive association between blood oxygenation level dependent (BOLD) response, which is the difference (placebo–oxytocin) in neural activation during presentation of faces in the left amygdala specified in arbitrary units (AU) and response time (RT) differences between face matching trials with and without prior application of oxytocin (*r* = 0.536, *R*^2^ = 0.288, *p* = 0.029)

In addition, the difference in neural activation between placebo and oxytocin trials during face-matching trials (difference: placebo—oxytocin, contrast: “faces”) in the left amygdala ROI significantly correlated with the difference in face-matching response times between oxytocin and placebo trials (difference: response times placebo—response times oxytocin) (*r* = 0.537, *p* = 0.029, *p*_FDR_ = 0.047 see Fig. 4), i.e. a larger oxytocin-induced attenuation in brain activation was associated with a larger difference in response times between oxytocin and placebo trials that is indicative of an association between modulated amygdala response and task-condition dependent differences in response times. That supports the notion that participants that show a differential pattern of amygdala activation between oxytocin and placebo trials also show a differential pattern of response times between task conditions. While associations for the right amygdala pointed towards an effect in the same direction, results did not yield statistical significance (*r* = 0.381, *p* = 0.100, *p*_FDR_ = 0.114). It should be noted that the correlation analyses are based on a rather small sample (*n* = 13) and should therefore be interpreted with all due caution and have to be replicated in larger samples. To support current results, we conducted leverage and outlier analyses using linear regression analyses in IBM SPSS Statistics for the associations between amygdala brain response and percent HDD (%HDD), as well as response times during face matching trials, because inspection of the scatter plots indicated that one participant might be an outlier. The leverage boundary set to 3*[*p*/*n*] (*p* = number of parameters and *n* = number of observations) and values were considered as outliers when they exceeded >  ± 1.96 and extreme outliers when they exceeded >  ± 3. None of the values had excessive leverage (see Supplementary figure S3 and S4), but one participant identified as outlier on the variable coding for %HDD and another on the variable coding for response times. We argue that in combination with the results of the leverage analyses, both participants did not identify as overly-influential and hence do not demand exclusion from correlation analyses, which in contrary would reduce the power (1-beta) of the correlation analyses by approximately 4%. Still, we performed additional analyses, excluding the respective participants. This rendered the association between oxytocin-induced left amygdala response attenuation and task reaction times insignificant (*p* = 0.352), while the association between right amygdala activation and %HDD remained significant (*p* = 0.013). While most of the findings of the correlation analyses are backed by correction for multiple comparisons and leverage and outlier analyses, the association between amygdala activation and task response times should be interpreted with caution, due to the small sample size.

## Discussion

Current data demonstrate a greater attenuation of amygdala activation by oxytocin selectively during the presentation of fearful and angry faces, compared to placebo, which was associated with lower alcohol craving and lower percentage of heavy drinking days. In line with this, animal studies showed that stress-enhanced fear-learning increases acquisition and maintenance of voluntary alcohol consumption in previously alcohol-naïve rats [19]. This supports the notion that a cross-talk between fear- and reward-processing systems might mediate the association between amygdala response to fear stimuli and alcohol craving. Based on preclinical data of Bowen et al. [2] that showed reduced alcohol consumption in rats after oxytocin application that was related to enhanced sociability and reduced anxiety, it can be speculated that a cross-talk between systems that process emotional states, social context, and drug craving mediates the observed effects. In line with this idea, oxytocin inhibited cocaine cue-induced anxiety in rats [21]. This finding suggests that oxytocin’s protective effect on cue-induced reinstatement of drug-seeking might relate to its anxiolytic effects. Corroborating this notion, animal studies showed that oxytocin application resulted in reduced alcohol ingestion and in addition prolonged reductions in anxious behavior [2]. Further, studies in humans showed that the effect of oxytocin was moderated by the individual attachment anxiety in AUD patients [20].

With regards to current findings, the selective modulation of amygdala activation during face-processing might reflect a neurobiological surrogate of oxytocin’s potential protective on negative emotion-driven drug seeking behavior and craving. A proposition that is also supported by preclinical data showing the relevance of the amygdala in mediating drug seeking behavior and relapse [6, 17]. Still, this hypothesis has to be validated in future studies.

In line with previous studies, we observed a positive BOLD response in the bilateral amygdala during face-matching trials of angry and fearful faces in the placebo condition [5, 15, 16]. BOLD response in the amygdala during face-matching trails was attenuated after oxytocin administration. This finding harmonizes with previous fMRI studies on the effects of oxytocin in healthy controls and patients with anxiety disorder that showed an attenuation of amygdala response after oxytocin application in both groups [5, 15, 16]. Current data also show that amygdala activation was near to zero during the shape-matching trials. This finding is in line with previous reports on the face-matching task and was expected, as the amygdala response should be specific to the emotional content of the stimulus and hence not generalize to the face-matching trials [26].

Task performance did not differ between medication conditions. This finding is in line with previous studies investigating a sample of healthy males using the same fMRI task and a similar cross-over design [15]. This might be explained by ceiling effects and due to a low variance of task performance. The finding of an association between a larger reduction in amygdala activation by oxytocin and task response times indicate that the modulation of amygdala activation is accompanied by changes to processing speed of facial stimuli.

Previous studies indicated a continuum for addiction-phenotypes from light to heavy social drinkers to alcohol addicted patients with higher levels of distress and craving [7]. Therefore, even though current results cannot be directly applied to clinical populations, it might facilitate the understanding of neurobiological and behavioral processes underlying alcohol consumption and addiction.

### Limitations

A limitation of the current study is the small sample size. This limited the capacity to detect small effects in the framework of the current study. Still, large effects, such as the interaction effects (medication × task-category) on functional brain activation (Eta^2^_leftAmygdala_ = 0.62, Eta^2^_rightAmygdala_ = 0.55) could be detected. This indicates that the main hypothesis, i.e. effects of oxytocin on brain activation during presentation of faces vs. forms could investigated with sufficient power. Still, further studies in larger samples are needed to replicate and validate current findings. There was a substantial range with regards to the age of participants in the current study. Although animal studies have provided evidence for age-related decreases in oxytonergic activity, the picture in humans is less clear. Only few studies specifically assessed the effects of aging on the human brain oxytocin system, most of whom relied on the analysis of post-mortem brain tissue. Only a handful of studies have investigated effects of aging on the human oxytocin system [13]. While evidence of reduced numbers of oxytonergic cells in the periventricular nucleus of older individuals was reported in one study [3], other studies failed to replicate significant effects of age on the number or size of oxytonergic cells [33]. While the within-subject cross over design reduced the risk of age bias in the current study, we still performed additional analyses of fMRI and behavioral data considering age as covariate. The significance of results did not change after controlling for age, supporting the robustness of our findings. While the incorporation of a sample of heavy social drinkers does not grant conclusions about processes in alcohol addiction, it might elucidate preceding stages.

## Conclusion

We are the first to show a specific and significant attenuation of the neural response to fearful face stimuli in the bilateral amygdala by oxytocin in a sample of moderate to heavy social drinkers. Additionally, we report a relation between the oxytocin-induced attenuation of amygdala activation during face-processing and alcohol craving and percentage of heavy drinking days. To conclude, oxytocin-induced attenuation of amygdala activation seems to be specific to processing negative face expressions and might represent a neurobiological substrate of oxytocin’s protective effect on craving.

## Electronic supplementary material

Below is the link to the electronic supplementary material.

Supplementary file1 (DOCX 3049 kb)
