# Supplementary material for: Oxytocin attenuates neural response to emotional faces in social drinkers: an fMRI study
Source: Eur Arch Psychiatry Clin Neurosci. 2020 Feb 19;271(5):873–82. doi: 10.1007/s00406-020-01115-0 (PMC8236029; doi:10.1007/s00406-020-01115-0)
Supplement: Supplementary file 1 — Supplementary file1 (DOCX 3049 kb) [file 406_2020_1115_MOESM1_ESM.docx]

**Supplementary Material**

Content:

**Figure S1 -** Consort Flow Diagram

**Figure S2 -** Functional magnetic resonance imaging (fMRI) Face-matching paradigm

**Figure S3 -** Depiction of the of leverage and outlier analyses for the linear regression analyses between amygdala brain response and percent heavy drinking days

**Figure S4 -** Depiction of the of leverage and outlier analyses for the linear regression analyses between amygdala brain response and response times during face matching trials

**Figure S1 - CONSORT Flow Diagram**

Responders (n= 46)

Inaccessible (n=18)

Excluded (n=3)

- Declined to participate (n=3)

Randomized (n=15)

Baseline (n=18)

Excluded (n=10)

- Not meeting inclusion criteria (n=5)
- Declined to participate (n=5)

Assessed for eligibility
(n=28)

**Analysis**

**T2**

Lost after T1 (n=0)

Lost after T1 (n=1) :

- Declined to participate (n=1)

**T1**

Lost before T1 (n=0)

Lost before T1 (n=0)

**Allocation**

Placebo

Scan 1

Oxytocin

45 min.

45 min.

2 weeks

Scan 2

Allocated to Group 2 (n=6)

Allocated to Group 1 (n=9)

Oxytocin

Scan 1

Placebo

45 min.

45 min.

2 weeks

Scan 2

Alcue task data for T1 and T2 (n=13):

- neurological incidental finding (n=1)

**Figure S2.**


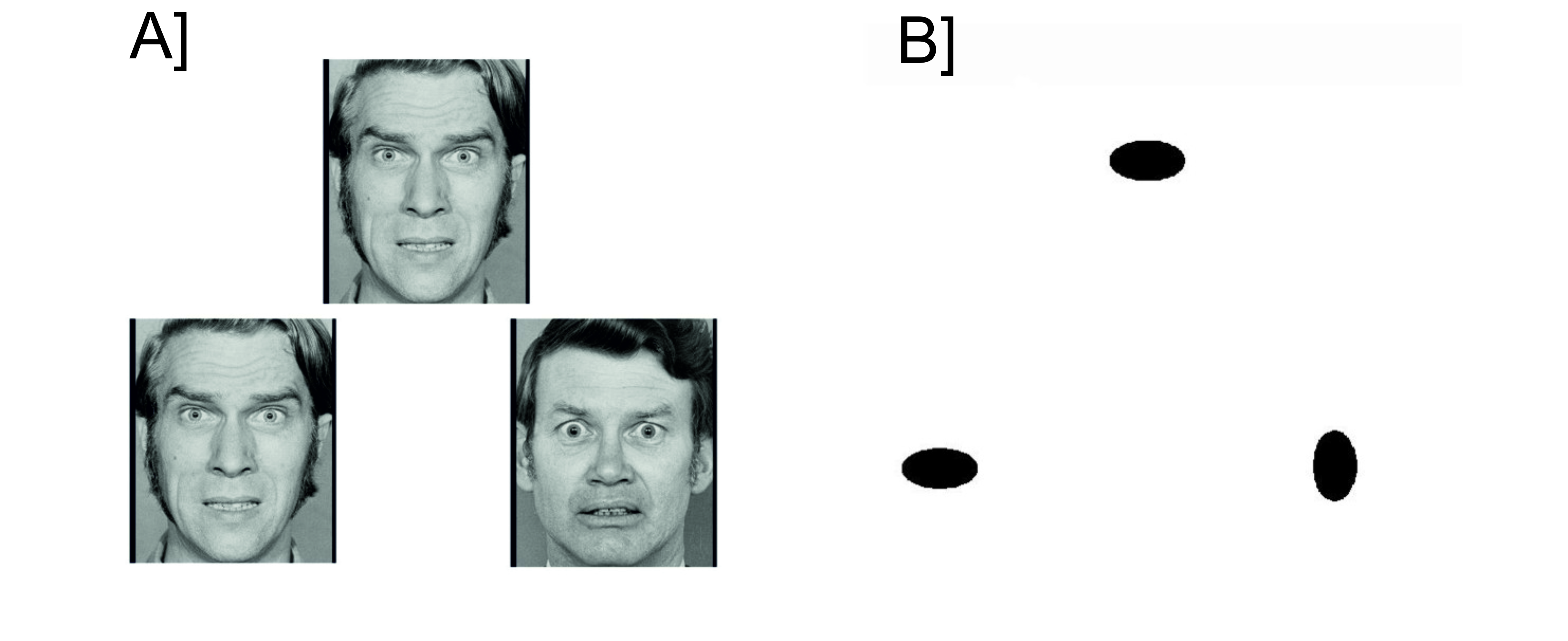


**Figure S2.** Experimental Face-matching paradigm. During the emotion task, **A]** subjects viewed a trio of faces and had to select which one of two faces (bottom) expressed the same emotion as a target face (top). An equal number of male and female faces was presented. During the control task, **B]** the subjects viewed a trio of geometric shapes and had to select which one of two shapes (bottom) was identical to a target shape (top).

**Figure S3**


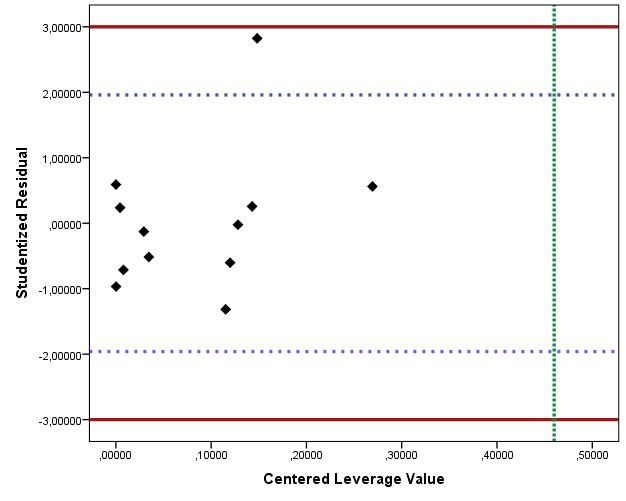


**Figure S3.** Depiction of the of leverage and outlier analyses for the linear regression analyses between amygdala brain response and percent heavy drinking days (%HDD). The leverage boundary (green dotted line) was set to 3*[p/n] (p = number of parameters and n = number of observations) and values were considered as outliers (blue dotted lines) when they exceeded > ± 1.96 and extreme outliers (red lines) when they exceeded > ± 3. None of the values had excessive leverage, but one participant identified as outlier on the variable coding for %HDD. and another on the variable coding for response times.

**Figure S4**


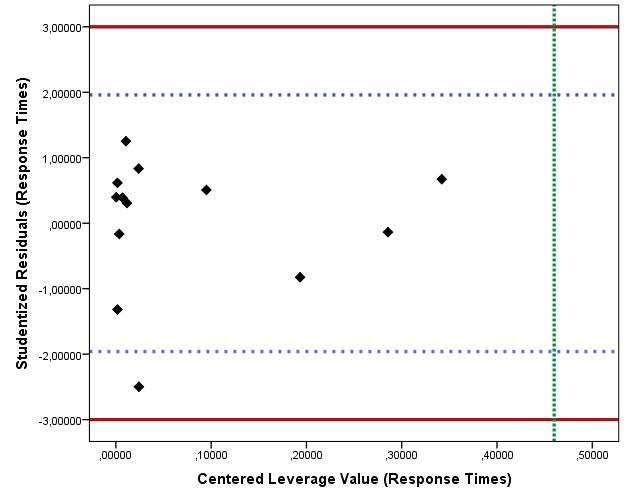


**Figure S4.** Depiction of the of leverage and outlier analyses for the linear regression analyses between amygdala brain response and response times during face matching trials. The leverage boundary (green dotted line) was set to 3*[p/n] (p = number of parameters and n = number of observations) and values were considered as outliers (blue dotted lines) when they exceeded > ± 1.96 and extreme outliers (red lines) when they exceeded > ± 3. None of the values had excessive leverage, but one participant identified as outlier on the variable coding for response times during face matching trials.
